# Analysis of Resident and Attending Physician End-of-Rotation Changeover Days and Association With Patient Length of Stay

**DOI:** 10.1001/jamanetworkopen.2023.4516

**Published:** 2023-03-23

**Authors:** Fizza Manzoor, Vaakesan Sundrelingam, Surain B. Roberts, Michael Fralick, Janice L. Kwan, Terence Tang, Adina S. Weinerman, Shail Rawal, Jessica J. Liu, Donald A. Redelmeier, Amol A. Verma, Fahad Razak, Lauren Lapointe-Shaw

**Affiliations:** 1 Division of General Internal Medicine, University of Toronto, Toronto, Ontario, Canada; 2 Li Ka Shing Knowledge Institute, St Michael’s Hospital, Toronto, Ontario, Canada; 3 Division of General Internal Medicine, Sinai Health, Toronto, Ontario, Canada; 4 Institute for Better Health, Trillium Health Partners, Toronto, Ontario, Canada; 5 Division of General Internal Medicine, Sunnybrook Health Sciences Centre, Toronto, Ontario, Canada; 6 Division of General Internal Medicine, University Health Network, Toronto, Ontario, Canada; 7 Division of General Internal Medicine, Unity Health, Toronto, Ontario, Canada; 8 Institute for Clinical Evaluative Sciences, Toronto, Ontario, Canada; 9 Toronto General Hospital Research Institute, Toronto, Ontario, Canada

## Abstract

**Importance:**

End-of-rotation resident physician changeover is a key part of postgraduate training but could lead to discontinuity in patient care.

**Objective:**

To test whether patients exposed to end-of-rotation resident changeover have longer hospital stays and whether this association is mitigated by separating resident and attending changeover days.

**Design, Setting, and Participants:**

This retrospective cohort analysis included adult patients admitted to general internal medicine. The changeover day was the same day (first Monday of month) for both resident and attending physicians until June 30, 2013 (preseparation period), and then intentionally staggered by 1 or more days after July 1, 2013 (postseparation period). This was a multicenter analysis at 4 teaching hospitals in Ontario, Canada, from July 1, 2010, to June 30, 2019. Data analysis was conducted from July 2022 to January 2023.

**Exposures:**

Patients were classified as changeover patients if the first Monday was a resident changeover day and as control patients if the first Monday was not a resident changeover day.

**Main Outcomes and Measures:**

The primary outcome was length of hospital stay. Secondary outcomes were transfer to critical care, in-hospital death, and rate of discharge per 100 patients on the index day.

**Results:**

Of 95 282 patients. 22 773 (24%; mean [SD] age, 67.8 [18.8] years; 11 156 [49%] female patients) were exposed to resident changeover, and 72 509 (76%; mean [SD] age, 67.8 [18.7] years; 35 293 [49%] female patients) were not exposed to resident changeover. Exposure to resident changeover day was associated with a slightly longer hospital stay compared with control days (0.20 [95% CI, 0.09-0.30] days; *P* < .001) and decreased relative risk of patient discharge on the index day (relative risk, 0.92; 95% CI, 0.86-1.00; *P* = .047). These associations were similar in the preseparation and postseparation periods. Resident changeover was not associated with an increased risk of transfer to critical care or in-hospital death.

**Conclusions and Relevance:**

In this study, a small positive association between exposure to resident physician changeover and length of hospital stay as well as reduced rate of discharge was found. These findings suggest that separating changeover days for resident and attending physicians may not significantly change these associations.

## Key Points

**Question:**

Do patients exposed to resident changeover have longer hospital stays, and does separating resident and attending physician changeover days modify this association?

**Findings:**

In this cohort study of 95 282 patients, exposure to end-of-rotation resident changeover was associated with an increase of 0.2 days in patients’ length of hospital stay and with a decreased likelihood of discharge on the day of changeover. These associations were not modified when the resident changeover days were separated from attending physician changeover days.

**Meaning:**

In this study, resident changeover was associated with marginally longer patient hospital stay that was not affected by separating changeover days for resident and attending physicians.

## Teaser

This cohort study examines whether patients exposed to end-of-rotation resident changeover have longer hospital stays and whether this association is mitigated by separating resident and attending changeover days.

## Introduction

Resident changeover, the process whereby residents leave one clinical rotation and begin the next, might lead to discontinuity in patient care. Resident physicians provide direct patient care, develop strong relationships with patients, and are essential to the functioning of inpatient teams.^1,2,3^ Changeover days may involve a steep learning curve as residents take over the care of unfamiliar and medically complex patients. The process of learning the nuanced clinical and social details of patients is time consuming, effortful, and imperfect.^4^ There are many predisposing challenges, including poor quality of written documentation, lack of structured verbal handover, and vague communication about rationale for clinical decisions.^5,6,7^ These factors may lead to a loss of diagnostic and therapeutic momentum resulting in additional investigations, longer hospital stays, health care costs, and potential complications.^8,9,10,11^

At academic centers, resident changeover occurs every 2 to 4 weeks; turnover in attending physicians poses an additional challenge to care continuity.^12,13^ Many interventions targeted at improving handover quality have reduced, but not eliminated, discontinuity in inpatient care.^14,15^ One strategy to encourage a safer end-of-rotation transition is to stagger the changeover days for residents and attending physicians, but the effectiveness of this intervention is unknown.^16^ The rationale is to overlap the transfer of care between an outgoing attending physician and an incoming team of residents to preserve relational and informational continuity. Our objective was to study whether resident physician changeover was associated with patient outcomes and whether this association was modified by a targeted intervention to separate resident and attending physician changeover days.

## Methods

### Study Setting and Overview

We conducted a multicenter study of patients admitted to or discharged from the general internal medicine service at 4 teaching hospitals affiliated with the University of Toronto in Ontario, Canada, from July 1, 2010, to June 30, 2019. The study protocol was approved by the Research Ethics Board of St Michaels’ Hospital including a waiver for direct patient consent due to the use of deidentified data, feasibility, minimal risk, and no direct effect on clinical care. We followed the Strengthening the Reporting of Observational Studies in Epidemiology (STROBE) reporting guideline for cohort studies.^17^

The University of Toronto has one of the largest internal medicine residency programs in North America and follows a monthly resident rotation changeover schedule.^18^ The 4 hospitals were core teaching sites for the internal medicine residency program: Mount Sinai Hospital, Toronto General Hospital, Toronto Western Hospital, and St Michael’s Hospital. At each site, the changeover day was the same day of the week (first Monday of each calendar month) for both resident and attending physicians until June 30, 2013 (preseparation period). Starting July 1, 2013, the changeover day remained the first Monday for residents and was moved to a different day for attending physicians (Wednesday or Thursday) with the aim of improving continuity (postseparation period). At each site, patients were exposed to a comparable level of trainee skillset, team size, ward structure, and supervision. The team structure was similar across all sites, typically composed of 1 attending physician, 1 senior resident (postgraduate year 2 or 3), 1 to 3 junior residents (postgraduate year 1 from internal medicine and other specialties), and 1 to 3 medical students (third and fourth year). The junior residents and medical students typically report to the senior resident as well as the attending physician. At the University of Toronto, residents in all direct-entry residency programs change rotation on the same day. In addition to the regular teaching teams, each site had 10% to 25% of its patients admitted to a nonteaching team during the study period. These were variably constructed. At 1 site, this was an attending physician, at times alone, at times accompanied by a single resident. Three sites had a nonteaching team with an attending, hospitalist fellows (who do not rotate monthly), and occasional residents. One site added a second nonteaching team in 2014, with an attending physician, a nurse practitioner, and a physician assistant.

### Patient Population

We used the General Medicine Inpatient Initiative (GEMINI) data set, which includes electronic administrative and clinical data about patients admitted to general internal medicine at several hospitals in Ontario.^19^ We included all adult patients (age 18 years or older) admitted to or discharged from the general internal medicine service at 1 of the 4 hospitals (eFigure 1 in Supplement 1). Patients whose hospital stay did not include the day of the week when resident changeover typically occurred were excluded. This day was Monday for 7 years and Tuesday for 1 year of the study period (2017-2018 academic year). We could not distinguish patients admitted to the nonteaching teams from patients admitted to teaching teams using the data available for this study.

### Exposure

We defined exposure according to the first Monday following the day of admission (index day). Patients were classified as changeover patients if the first Monday was a resident changeover day and as control patients if the first Monday was not a resident changeover day. This design minimized bias by ensuring that both groups were exposed to a similar number of weekdays, weekend days, and total admitted days before exposure assignment.^20^ Only the first Monday was used to assign exposure as the mean length of stay of internal medicine patients in our setting is typically less than 1 week.^21^ We also estimated that diagnostic and treatment delays would likely have a greater impact on patient outcomes earlier in the hospitalization, when the acuity of care is typically greatest.^22^ One exception for exposure assignment was the academic year from July 2017 to June 2018, when the day of resident rotation changeover was briefly changed to Tuesday; for this year only, the first Tuesday following admission was used to assign exposure.

### Outcomes

The primary outcome was length of hospital stay from the index day (changeover day or control day) to discharge, up to 30 days. Measurement of the length of hospital stay was truncated at 30 days because most hospital admissions are shorter than 1 month (admissions longer than 30 days accounted for only 3.9% of admissions in our study) and we expected that care discontinuity in the first week of admission would not affect clinical outcomes beyond 30 days.

Secondary outcomes were transfer to a critical care within 7 days of index day, in-hospital death within 7 days of index day, and the rate of discharge on the index day (measured as the number of alive discharges per 100 admitted patients). A discharge on the index day was defined as a patient discharged between 8:00 am and midnight. The outcome of in-hospital death excluded deaths attributed to suicide and included deaths attributed to medical assistance in dying (0.13% of all in-hospital deaths).

### Baseline Characteristics

We included baseline patient characteristics and other variables that might affect the length of hospital stay. These included patient demographics (age, sex, neighborhood income quintile),^23^ Charlson Comorbidity Index score,^24,25^ Laboratory-based Acute Physiology Score,^26^ hospital admission in the 30 previous days,^27^ day of admission (weekday vs weekend or holiday),^20^ most responsible discharge diagnosis (if 1 of 10 most common discharge diagnoses),^28^ number of days in hospital prior to index day, month of admission,^29^ calendar year of admission,^30^ hospital general internal medicine census on index day,^31^ and hospital site.^22^ The general internal medicine capacity ratio was calculated for both groups to compare inpatient census on index day and was derived by dividing the total census on the index day by the annual median census.

### Statistical Analysis

#### Primary Analysis

We first compared unadjusted baseline characteristics between patients exposed to changeover and control index day using standardized mean difference (SMD), in which a difference of greater than 10% (0.10) was considered a meaningful difference.^32^ Clinical care may have changed during the study period and might create time trends that could affect our findings.^33,34,35^ For this reason, we plotted the mean length of stay by month and by year. We also assessed for underlying secular trends by plotting the monthly unadjusted relative risk over time and assessing for a linear trend.^36^ Because we did not identify underlying secular trends, we proceeded with a pre-post comparison.

For the primary analysis, we used a negative binomial regression to measure the adjusted association between exposure to resident changeover and length of hospital stay. We then used an interaction term to test whether the separation of changeover days (preseparation, July 1, 2010, to June 30, 2013; postseparation, July 1, 2013, to June 30, 2019) affected the association between exposure to changeover and length of stay. The analysis was adjusted for all measured baseline characteristics (except for admission month and year), variables that were selected a priori for their potential to confound the outcomes. Data analysis was conducted from July 2022 to January 2023.

#### Sensitivity Analyses

To address concerns that any potential handover effect was diluted by the long total hospital stay of some inpatients, we restricted the study population to those with hospital length of stay less than 2 weeks. This 2-week cutoff was used as an exposure in the first week of admission would intuitively be most likely to have a measurable impact on clinical outcomes in the week following, and we expected that the consequences of the exposure would be weaker as time passed after the initial exposure event.

#### Secondary Outcome Analyses

We used logistic regression to model the 7-day transfer to critical care and 7-day in-hospital death outcomes. We used unadjusted negative binomial regression to compare the rate of alive discharges per 100 patients on changeover days (offset term) compared with control days. The analysis of discharge rate was unadjusted because it evaluated data that was aggregated at the hospital level (not patient level).

We reported all regression outputs as adjusted relative risks and odds ratios with 95% CIs. In addition, we also reported the adjusted difference in days for length of hospital stay with 95% CIs using average marginal effects (where all covariates assume their naturally observed distributions), using the margins package version 0.3.26 in R software.^37,38^ All analyses were performed in R version 3.6.2 (R Project for Statistical Computing). Hypothesis testing was done at a 2-tailed significance threshold of *P* < .05.

## Results

A total of 95 282 patients were included in our study cohort: 22 773 (24%; mean [SD] age, 67.8 [18.8] years; 11 156 [49%] female patients) experienced a resident changeover in the first week of admission, and 72 509 (76%; mean [SD] age, 67.8 [18.7] years; 35 293 [49%] female patients) did not. The 2 groups had similar distributions of baseline characteristics (Table 1). Most patients in both groups had not been admitted to hospital in the preceding month (patients with admission in past month, changeover patients: 2230 [9.8%]; control patients: 7225 [10.0%]; SMD, 0.01), and approximately half spent less than 2 days in hospital prior to the index day (changeover patients: 11 966 [52.5%]; control patients: 38 562 [53.2%]; SMD, 0.02). The mean (SD) general internal medicine capacity ratio was similar on changeover and control days (1.03 [0.10] vs 1.04 [0.10]; SMD, 0.08). The 3 most common discharge diagnoses were also the same in both groups: pneumonia, bacterial infections, and congestive heart failure (SMD, 0.04).

**Table 1.** Baseline Characteristics

Characteristic	Patients, No. (%)		Standardized mean difference^a^
	Control (n = 72 509)	Changeover (n = 22 773)	
Age, y			
Mean (SD)	67.8 (18.7)	67.8 (18.8)	0.00
Median (range)	71.0 (18.0-119)	71.0 (18.0-118)	
Sex			
Female	35 293 (48.7)	11 156 (49.0)	0.01
Male	37 216 (51.3)	11 617 (51.0)	
Neighborhood income quintile^b^			
1, Lowest	24 742 (34.1)	7717 (33.9)	0.01
2	12 451 (17.2)	3912 (17.2)	
3	10 562 (14.6)	3356 (14.7)	
4	8096 (11.2)	2516 (11.0)	
5, Highest	6222 (8.6)	1932 (8.5)	
Missing	10 436 (14.4)	3340 (14.7)	
Charlson Comorbidity Index at admission			
0	43 678 (60.2)	13 631 (59.9)	0.01
1	8997 (12.4)	2843 (12.5)	
2	10 854 (15.0)	3494 (15.3)	
≥3	8980 (12.4)	2805 (12.3)	
Laboratory-based Acute Physiology Score^c^			
Mean (SD)	17.4 (15.3)	17.4 (15.3)	0.00
Median (range)	15.0 (0-165)	15.0 (0-134)	
Admission to general internal medicine in the last 30 d			
Yes	7225 (10.0)	2230 (9.8)	0.01
No	64 349 (88.7)	20 280 (89.1)	
Missing	935 (1.3)	263 (1.2)	
Hospital site			
Mount Sinai Hospital	17 429 (24.0)	5339 (23.4)	0.02
St Michael’s Hospital	17 215 (23.7)	5529 (24.3)	
Toronto General Hospital	19 241 (26.5)	6045 (26.5)	
Toronto Western Hospital	18 624 (25.7)	5860 (25.7)	
Days in hospital prior to index day			
0-1	38 562 (53.2)	11 966 (52.5)	0.02
2-4	27 654 (38.1)	8725 (38.3)	
5-6	6293 (8.7)	2082 (9.1)	
Day of admission			
Weekday	49 037 (67.6)	15 621 (68.6)	0.02
Weekend or holiday	23 472 (32.4)	7152 (31.4)	
Admission month			
January	6737 (9.3)	2179 (9.6)	0.14
February	5592 (7.7)	1825 (8.0)	
March	5613 (7.7)	1633 (7.2)	
April	5976 (8.2)	2112 (9.3)	
May	5782 (8.0)	2457 (10.8)	
June	6062 (8.4)	1824 (8.0)	
July	6268 (8.6)	1850 (8.1)	
August	6041 (8.3)	1956 (8.6)	
September	6213 (8.6)	1558 (6.8)	
October	6108 (8.4)	2033 (8.9)	
November	5806 (8.0)	1776 (7.8)	
December	6311 (8.7)	1570 (6.9)	
Admission year			
2010	3609 (5.0)	990 (4.3)	0.08
2011	7430 (10.2)	2100 (9.2)	
2012	7612 (10.5)	2176 (9.6)	
2013	7534 (10.4)	2416 (10.6)	
2014	8268 (11.4)	2792 (12.3)	
2015	8292 (11.4)	2690 (11.8)	
2016	7999 (11.0)	2626 (11.5)	
2017	8729 (12.0)	2627 (11.5)	
2018	8852 (12.2)	2797 (12.3)	
2019	4184 (5.8)	1559 (6.8)	
General internal medicine capacity ratio			
Mean (SD)	1.04 (0.10)	1.03 (0.10)	0.08
Median (range)	1.04 (0.67-1.37)	1.04 (0.74-1.36)	

^a^ Standardized mean difference significant at 0.10.

^b^ Quintile 1, annual income less than $40 797; 2, annual income $40 797 to $48 639; 3, annual income $48 639 to $55 044; 4, annual income $55 044 to $64 581; 5, annual income greater than $64 581.

^c^ The Laboratory-based Acute Physiology Score consists of abnormal laboratory test results and is used to predict inpatient mortality. It is scored from 0 to 256, with a higher score representing increased risk of mortality.

### Length of Hospital Stay

The mean (SD) length of hospital stay after the index day was 6.7 (7.3) days for patients exposed to resident changeover and 6.5 (7.3) days for patients not exposed to resident changeover (SMD 0.03) (Table 2). The unadjusted association between changeover and length of stay did not demonstrate any secular trend (eFigures 2 and 3 in Supplement 1). After adjustment and over the entire study period, resident changeover day was associated with a slightly longer hospital stay compared with control days (adjusted relative risk [aRR], 1.05; 95% CI, 1.02-1.08; *P* < .001) equal to an absolute adjusted difference of 0.20 days per patient (95% CI, 0.09-0.30 days; *P* < .001) (Table 3). This association was not significantly different in the preseparation and postseparation periods (preseparation aRR, 1.05 [95% CI, 1.02-1.08]; postseparation aRR, 1.02 [95% CI, 1.00-1.04]; *P *for interaction = .10).

**Table 2.** Clinical Outcomes During Study Period

Outcome	Patients, No. (%)		
	Preseparation	Postseparation	Overall
Length of hospital stay, mean (SD), d	6.7 (7.4)	6.5 (7.3)	6.5 (7.3)
Critical care transfer within 7 d	1058 (3.7)	4143 (6.2)	5201 (5.5)
Death within 7 d	818 (2.9)	1530 (2.3)	2348 (2.5)
Discharge alive on index day, mean (SD), discharges per 100 patients	11.38 (6.9)	11.72 (6.5)	11.61 (6.7)

**Table 3.** Adjusted Association Between Exposure to Changeover and Clinical Outcomes

Outcome	Preseparation	Postseparation	Overall	*P* value for interaction
Length of hospital stay, aRR (95% CI)	1.05 (1.02-1.08)	1.02 (1.00-1.04)	1.05 (1.02-1.08)	.10
Critical care transfer within 7 d, aOR (95% CI)	1.05 (0.90-1.22)	0.93 (0.85-1.02)	1.02 (0.88-1.19)	.38
Death within 7 d, aOR (95% CI)	0.90 (0.74-1.07)	0.98 (0.87-1.12)	0.88 (0.74-1.06)	.33
Discharge alive on index day, RR (95% CI)	0.93 (0.86-1.00)	0.96 (0.92-1.00)	0.92 (0.86-1.00)	.46

Abbreviations: aOR, adjusted odds ratio; aRR, adjusted relative risk; RR, relative risk.

### Sensitivity Analysis: Length of Hospital Stay

Sensitivity analysis of patients with total stay less than 2 weeks (82 584 patients; 87% of total sample size) found that resident changeover day remained associated with longer hospital stay compared with control days (aRR, 1.05; 95% CI 1.02-1.07, *P* < .001), equal to an absolute adjusted difference of 0.13 days (95% CI, 0.08 to 0.18 days; *P* < .001). This association was not significantly different in the preseparation and postseparation periods (interaction *P* = .15).

### Secondary Outcomes

Transfer to critical care within 7 days of the index day occurred in 5.5% of admissions for both groups (5201 patients), with no significant association with exposure to resident changeover day (adjusted odds ratio [aOR], 1.02; 95% CI, 0.88-1.19, *P* = .79). There was no difference in the preseparation and postseparation periods (interaction *P* = .38).

In-hospital death within 7 days of index day occurred in 2.4% of admissions for patients exposed to resident changeover (537 patients) and in 2.5% for patients not exposed to resident changeover (1811 patients). There was no significant association with exposure to resident changeover day (aOR, 0.88; 95% CI, 0.74 to 1.06; *P* = .17), and no difference in the preseparation and postseparation periods (*P *for interaction = .33).

The mean (SD) number of discharges per 100 patients was 10.9 (4.9) discharges on the day of resident changeover and 11.6 (6.7) discharges on a control weekday (SMD, 0.12). Over the entire study period, exposure to resident changeover day was associated with a decreased likelihood of patient discharge on the changeover day compared with control days (RR, 0.92; 95% CI, 0.86-1.00, *P* = .047). This association was not significantly different in the preseparation and postseparation periods (*P *for interaction = .46).

## Discussion

We studied more than 95 000 patients over 9 years to test whether separating resident and attending physician changeover improved patient outcomes. We found that resident changeover was associated with a marginally longer hospital stay and a lower rate of discharge compared with nonchangeover days. The separation of resident and attending physician changeover days did not significantly attenuate these associations. Fortunately, there was no association between resident changeover and increased risk of death or transfer to critical care within 7 days after changeover.

Our study adds to prior research on physician transitions by showing that resident changeovers might be associated with longer hospital stay.^39^ The reasons for slightly increased length of stay may include hesitation to discharge an unfamiliar patient on the first day, loss of diagnostic momentum, or lack of time to discharge safely when acclimating to a new group of patients. Given that all residents in entry-level programs switch rotations on the same day, it is also possible that delays in obtaining imaging or consultation contributed to the increase in length of stay in the changeover-exposed group. A modest increase in length of hospital stay by 0.13 to 0.20 days per patient (sensitivity analysis vs overall analysis), if multiplied by the typical number of patients exposed to changeover in our setting annually (0.24 × 10 500 admissions per year at 4 study sites), would translate to an additional 327 to 504 bed-days annually. In Ontario, where hospitals regularly operate above funded capacity, even a small increase in demand for hospital bed-days is concerning.^40^

Most prior research on resident changeovers has focused on the annual changeover in July (start of residency in North America) or daily changeover (short vs long working shifts), with few studies examining end-of-rotation changeover.^41,42,43,44,45^ Some studies have shown that patients admitted at the time of resident changeover had longer hospital stay and that patients discharged within a week of resident changeover had higher mortality rates.^46,47,48,49^ In contrast to our findings, one study found that resident changeover had no association with discharge rate from hospital.^50^ Some prior estimates of length of stay have been as long as 1.6 additional days; however, our estimate may be lower (0.20 days) because of the additional steps we took to reduce bias.^44^ In contrast to our design, many previous studies did not assign the exposure based on an index day of the week; as a result, patients with longer length of stay would have more chances to be exposed to changeover, introducing reverse causation.^43,44,45,46,47^

End-of-rotation resident changeover introduces discontinuity into patient care, which can be compounded by attending physician changeover. Eliminating changeover is not feasible because current postgraduate training requires exposure to multiple specialties, diverse patient groups, and clinical variety to develop competency.^51^ Furthermore, learning to efficiently care for unfamiliar patients is an important skillset for future practice. Separating changeover days is an intuitive, inexpensive, and adaptable solution to increase care continuity. Our findings should not be used to justify the elimination of separate changeover days, which may have other benefits for patients and trainee experience, such as improved communication, patient comfort, sense of team belonging, and support at the start of a new rotation.^52^

### Limitations

To our knowledge, our study is the first to evaluate the outcomes of a widely applied intervention to improve end-of-rotation changeover: separating resident and attending physician changeover days; however, our study several limitations. We took steps to minimize upward bias in our estimates of the association of resident changeover with length of stay, but several elements could have biased our findings toward the null. First, a minority of patients at 3 of our 4 included hospitals were admitted to nonteaching teams, although our data sources could not identify which patients these were. Resident changeover would be expected to have minimal effect on these teams, although many have hospitalist fellows who sometimes change over, often less frequently than residents.^53^ Second, continuity of interprofessional teams and residents on rotations longer than 1 month may also have biased our estimates toward the null. Any downward bias on the association between resident changeover and length of stay also reduced our ability to detect a significant change on changeover separation. Our findings have not ruled out a benefit of separating changeover days; however, any benefit is likely to be small.

Additionally, the available data did not capture other outcomes related to learner and patient experience that are likely affected by maintaining continuity of care during changeover.^54^ Furthermore, we did not explore the outcomes of attending physician changeover in isolation.

## Conclusions

In this study, exposure to resident changeover was associated with a slightly increased length of patient stay; however, this association was not modified by the separation of resident and attending changeover days. The patient and learner continuity afforded by the separation of resident and attending changeover may have other benefits that should be explored in future research.

## Notes

Supplement 1. **eFigure 1.** Study Flowchart **eFigure 2.** Mean Length of Stay Over Time **eFigure 3.** Time Series Plot

Supplement 2.
Data Sharing Statement
