# Supplementary material for: Analysis of Resident and Attending Physician End-of-Rotation Changeover Days and Association With Patient Length of Stay
Source: JAMA Netw Open. 2023 Mar 23;6(3):e234516. doi: 10.1001/jamanetworkopen.2023.4516 (PMC10037142; doi:10.1001/jamanetworkopen.2023.4516)
Supplement: Supplement 1. — eFigure 1. Study Flowchart eFigure 2. Mean Length of Stay Over Time eFigure 3. Time Series Plot [file jamanetwopen-e234516-s001.pdf]

Supplemental Online Content

Manzoor F, Sundrelingam V, Roberts SB, et al. Analysis of resident and attending physician end-of-rotation changeover days and association with patient length of stay. *JAMA Netw Open.* 2023;6(3):e234516. doi:10.1001/jamanetworkopen.2023.4516

eFigure 1. Study Flowchart

eFigure 2. Mean Length of Stay Over Time

eFigure 3. Time Series Plot

This supplemental material has been provided by the authors to give readers additional information about their work.

© 2023 Manzoor F et al. *JAMA Network Open.*

eFigure 1. Study Flowchart
A study flow summarizes selection of the patient cohort after application of the defined inclusion and exclusion criteria.

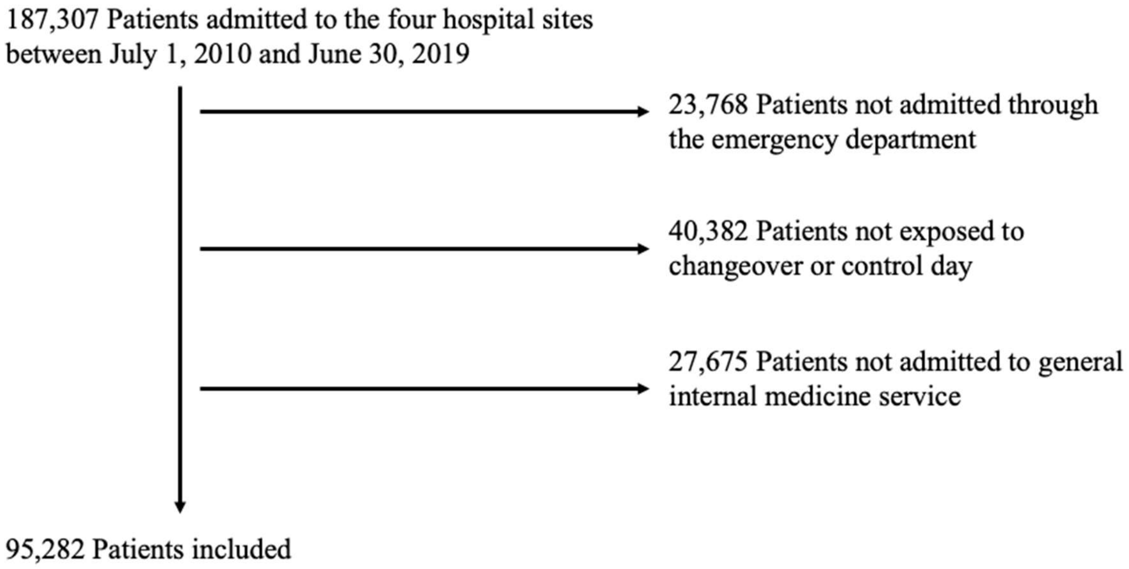

© 2023 Manzoor F et al. *JAMA Network Open.*

eFigure 2. Mean Length of Stay Over Time

Mean length of stay as calculated and plotted over time. The plot shows time on the X-axis (admission year) and mean length of stay on the Y-axis. Each dark blue circular dot represents the mean length of stay by month, and light blue circular dot represents the mean length of stay by year.

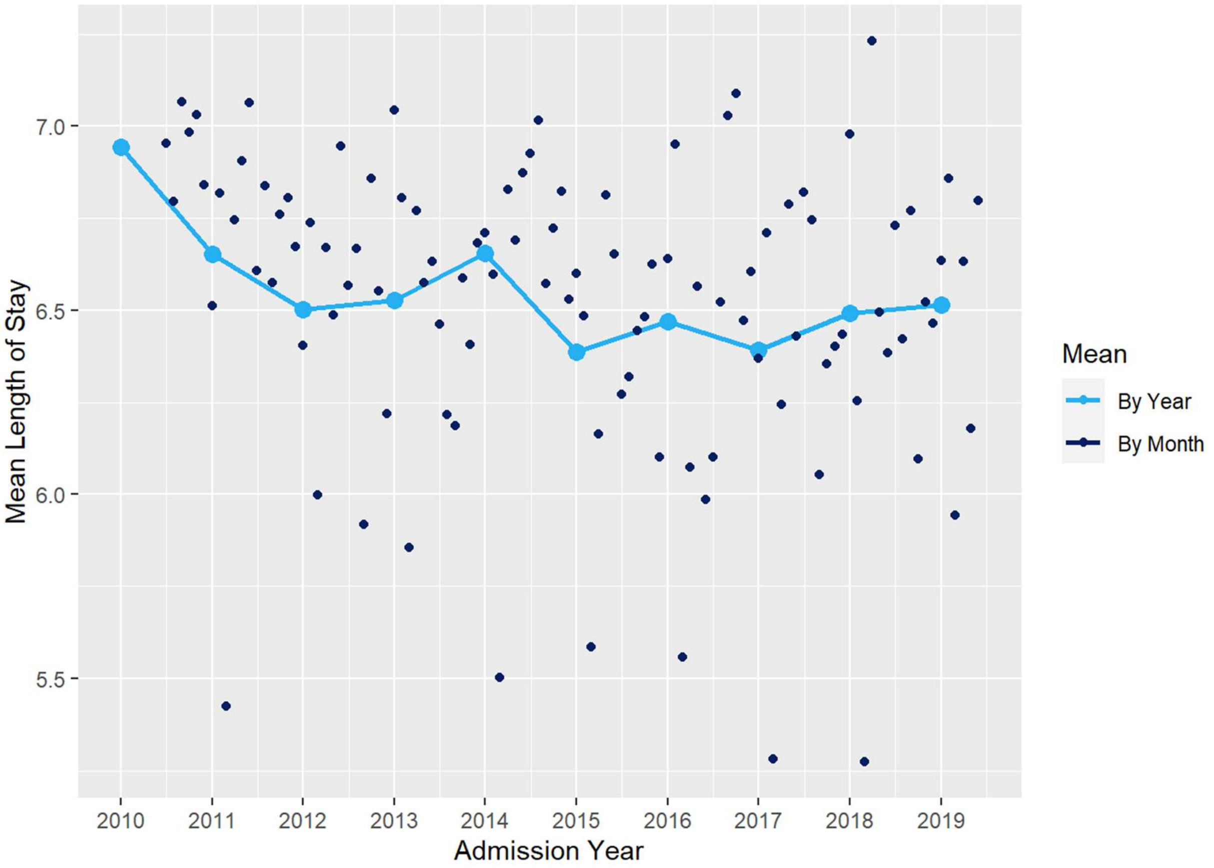

© 2023 Manzoor F et al. *JAMA Network Open.*

eFigure 3. Time Series Plot

Monthly unadjusted relative risks for length of stay were calculated for the months preceding July 2013 and the months following July 2013 over the study period. A linear regression was used to test for association between the monthly relative risks and handover block (time). Results showed no significant correlation between the monthly relative risks over time (p = 0.265).

The time series plot shows time on the X-axis (monthly handover block) and relative risk on the Y-axis. Each circular dot represents the unadjusted relative risk (for length of stay) and the accompanying vertical line represents the 95% confidence interval. Two blue crossbars are centered at the unadjusted relative risk for the period pre-and post-separation of changeover days. The upper and lower limits of the crossbars represent the upper and lower bounds for their respective 95% confidence intervals. Two thick solid grey slopes show a piecewise linear regression fit to the data with a breakpoint on July 2013, representing the date of handoff separation. For reference, a dashed horizontal grey line is drawn at RR = 1.0 (the null hypothesis) and a dashed vertical navy line is drawn at July 2013.

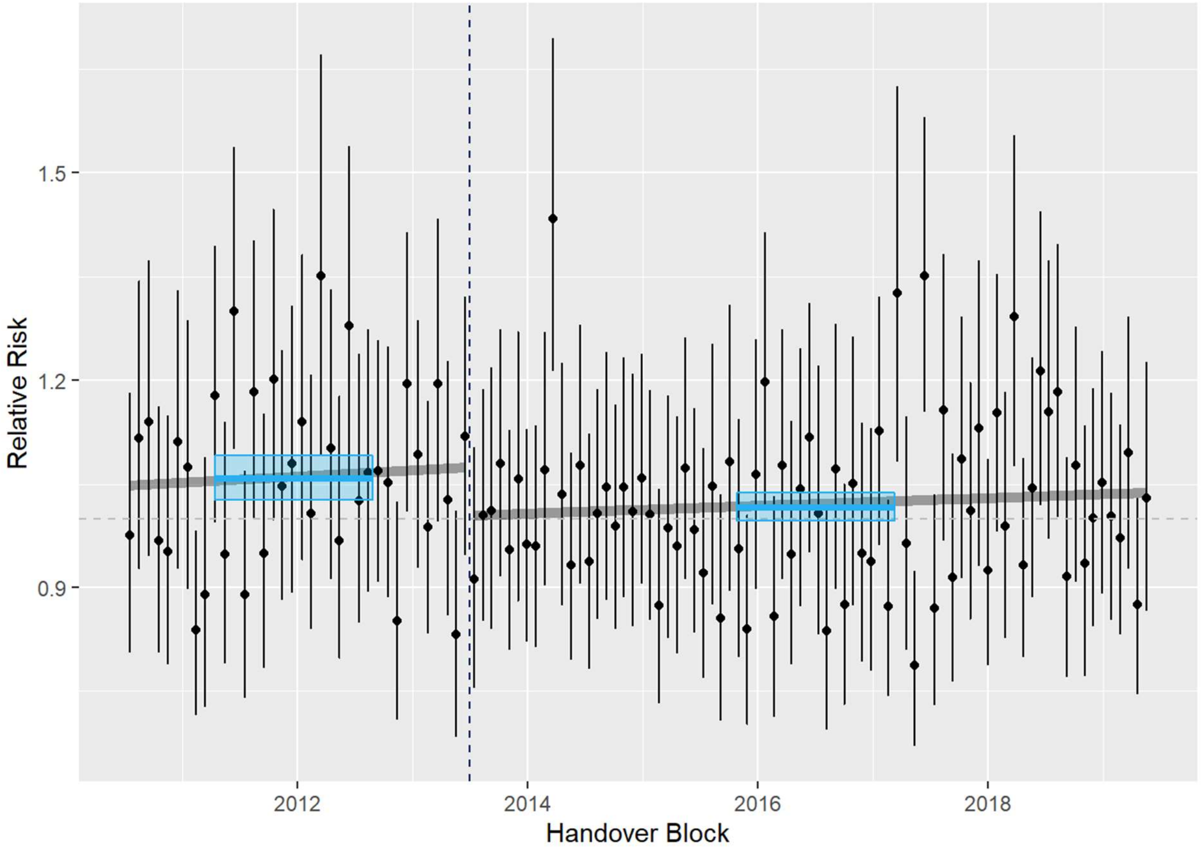

© 2023 Manzoor F et al. *JAMA Network Open.*
