# Supplementary material for: Analysis of Resident and Attending Physician End-of-Rotation Changeover Days and Association With Patient Length of Stay
Source: JAMA Netw Open. 2023 Mar 23;6(3):e234516. doi: 10.1001/jamanetworkopen.2023.4516 (PMC10037142; doi:10.1001/jamanetworkopen.2023.4516)
Supplement: Supplement 2. — Data Sharing Statement [file jamanetwopen-e234516-s002.pdf]

Data Sharing Statement

Manzoor. Analysis of Resident and Attending Physician End-of-Rotation Changeover Days and Association With Patient Length of Stay. *JAMA Netw Open*. Published March 23, 2023. doi:10.1001/jamanetworkopen.2023.4516

Data
Data available: No
